# Primary bacteriemia and catheter related bloodstream infection in patients admitted to ICU. risk factors associated with mortality. ENVIN-HELICS registry data

**DOI:** 10.1186/2197-425X-3-S1-A889

**Published:** 2015-10-01

**Authors:** X Nuvials, M Palomar, F Alvarez-Lerma, P Olaechea, S Otero, S Uriona, M Catalán, R Gimeno, MP Gracia, I Seijas, ENVIN-HELICS

**Affiliations:** , Intensive Care Medicine, Hospital Universitari Arnau de Vilanova. IRB Lleida, Lleida, Spain; Intensive Care Medicine, Hospital Universitari Arnau de Vilanova. IRB Lleida, Lleida, Spain; Intensive Care Medicine, Hospital del Mar, Barcelona, Spain; Intensive Care Medicine, Hospital de Galdakao, Galdako, Spain; Medicina Preventiva, Hospital Universitari Vall d'Hebron, Barcelona, Spain; Intensive Care Medicine, Hospital 12 de Octubre, Madrid, Spain; Intensive Care Medicine, Hospital La Fe, Valencia, Spain; Intensive Care Medicine, Hospital de Cruces, Barakaldo, Spain

## Introduction

Primary bacteriemia (PB) and catheter related bloodstream infections(CRBI) have been associated with higher mortality in critically ill patients.

## Objectives

To study the risk factors associated with mortality in patients admitted to ICU with PB-CRBI.

## Methods

Prospective, observational, multicenter and voluntary enrollment study (Spanish registry ENVIN-HELICS) [1]. All patients admitted to ICU for > 24 hours between 1st April and 30th June along the period from 2006 to 2013 were included. All episodes of PB-CRBI were recorded during the follow-up. BP-CRBI was defined according HELICS-IPSE definitions [2]. Demographic data, risk factors, ICU mortality and the presence of any non healthcare related infection were recorded. Univariate analysis was done using Chi-square test and variables found to be significant (p < 0,05) were included in a multivariate logistic regression model. p value < 0,05 was considered statistical significant.

## Results

Among 129,037 patients admitted to ICU, 58,706 infections were recorded, of whom 4,479 (7.6%) were PB-CRBI. Table 1 shows risk factors found to be associated with mortality in patients with PB-CRBI in the multivariate analysis.

**Table 1:** Table 1

Variable	Odds ratio	IC 95%	p
Age	1,01	[1,01-1,02]	< 0.05
Sex	1,06	[0,86-1,31]	NS
APACHE II	1,06	[1,05-1,08]	< 0.05
Elective surgery	0,67	[0,45-1,00]	NS
Emergency surgery	0,82	[0,60-1,11]	NS
Trauma	0,46	[0,27-0,77]	< 0.05
Continuous renal replacement	2,36	[1,87-2,98]	< 0.05
Non healthcare related infection	1,40	[1,14-1,71]	< 0.05

## Conclusions

The severity of illness on admission, the use of continuous renal replacement systems, as well as the presence of non healthcare related infections, are risk factors independently associated with mortality in patients with PB-CRBI admitted to ICU.

## References

[1] [http://hws.vhebron.net/envin-helics/]

[2] Hospital in Europe Link for Infection Control through Surveillance (HELICS). Version 6.1. Sep. 2004. Surveillance of Nosocomial Infections in Intensive Care Units. [http://www.ecdc.europa.eu/IPSE/protocols/icu_protocol.pdf]

